# Survival Outcomes with Photodynamic Therapy, Chemotherapy and Radiation in Patients with Stage III or Stage IV Non-Small Cell Lung Cancer

**DOI:** 10.3390/cancers13040803

**Published:** 2021-02-15

**Authors:** Sumedha Chhatre, Anil Vachani, Ron R. Allison, Ravishankar Jayadevappa

**Affiliations:** 1 Perlman School of Medicine, University of Pennsylvania, Philadelphia, PA 19104, USA; rasu@pennmedicine.upenn.edu (S.C.); avachani@pennmedicine.upenn.edu (A.V.); 2 Leonard Davis Institute of Health Economics, Perlman School of Medicine, University of Pennsylvania, Philadelphia, PA 19104, USA; 3 Department of Radiation Oncology, Federal Medical Center, Butner, NC 27509, USA; ronrallison@gmail.com

**Keywords:** non-small cell lung cancer (NSCLC), photodynamic therapy (PDT), National Cancer Database (NCDB), radiation therapy, chemotherapy, mortality

## Abstract

**Simple Summary:**

The association between photodynamic therapy (PDT) and mortality in lung cancer patients remains unclear. We studied the association between PDT and mortality in patients with stage III/IV non-small cell lung cancer (NSCLC) using the National Cancer Database (NCDB) between 2004 and 2016. From the NCDB, we identified patients whose treatment code was ablation (including PDT). From Medicare and Medicaid data between 2000 and 2013, we identified NSCLC patients receiving PDT and used these to confirm PDT treatment. We assessed the association between PDT and mortality. Study groups consisted of 147 patients with PDT + radiation + chemotherapy, 227,629 with radiation + chemotherapy, 106,667 with radiation therapy alone and 122,193 with chemotherapy alone. Compared to the radiation alone group, the PDT group and radiation with chemotherapy group had lower risk of mortality (50% and 53% lower, respectively). Among the NSCLC patients with stage III or stage IV disease not eligible for surgery, the addition of PDT to chemotherapy and radiation therapy offers survival benefit over radiation therapy alone.

**Abstract:**

Data regarding the association between photodynamic therapy (PDT) and mortality in lung cancer patients are limited. We analyzed the association between PDT and mortality in patients with stage III or IV non-small cell lung cancer (NSCLC) using data from the National Cancer Database (NCDB) between 2004 and 2016. From the NCDB, we identified patients receiving laser ablation/cryosurgery or local tumor destruction/excision (which includes PDT). From Medicare and Medicaid claims between 2000 and 2013, we identified NSCLC patients receiving PDT and those receiving bronchoscopy, then used these to confirm the PDT treatment. From NCDB, we extracted NSCLC patients who received radiation with chemotherapy, radiation alone or chemotherapy alone. We used survival analysis to determine the association between PDT and mortality. Between 2004 and 2016, 457,556 NSCLC patients with stage III or stage IV were identified, of which 147 received PDT with radiation and chemotherapy, 227,629 received radiation with chemotherapy, 106,667 had radiation therapy alone and 122,193 received chemotherapy alone. Compared to the radiation alone group, the PDT group and radiation with chemotherapy group had lower hazard of mortality (50% and 53% lower, respectively). Among the NSCLC patients with stage III or stage IV disease, the addition of PDT to radiation therapy offers survival benefit over radiation therapy alone.

## 1. Introduction

In 2020, lung cancer was expected to be one of the top three cancers for both men and women in the US [1]. A total of 228,820 new cases of lung cancer were diagnosed (116,300 in men and 112,520 in women), and approximately 135,720 deaths from lung cancer occurred (72,500 in men and 63,220 in women) in the year 2020 [1]. Lung cancer is generally classified as small-cell or non-small cell lung cancer (NSCLC), with the latter being more frequent. Patients with NSCLC tend to be older and therefore experience several comorbidities [2]. Severity of NSCLC has resulted in the development of multiple treatment options, both therapeutic and palliative. Lung cancer screening using low-dose CT scan imaging has demonstrated improved survival, as many screens detect relatively early stage of disease, with a high potential for cure.

Although surgical resection offers a strong opportunity for cure in early stage NSCLC cases, the presence of comorbid conditions can deem a patient to be medically inoperable. One option for these patients is radiosurgery, which is the precise delivery of radiation therapy to a limited tumor volume [3,4]. Additionally, other types of ablation therapies, such as photodynamic therapy (PDT), are promising. Used alone or as part of multi-modal therapy, PDT can help decrease the probability of disease progression and/or mortality in NSCLC patients who present with high-risk localized disease, as well as for locally advanced, recurrent, or metastatic disease [5,6,7,8,9].

Late-stage disease mostly renders patients unsuitable to receive curative surgery, largely due to extent of disease. Treatments, such as radiation therapy, chemotherapy, immunotherapy, and ablation therapies, such as PDT, cryotherapy, argon plasma coagulation and ND:YAG laser, either alone or in combination, have demonstrated improved outcomes [5,6,7,8,9,10,11]. Among NSCLC patients, many types of ablation therapies, either as monotherapy or as part of the multimodal treatment approach, have shown favorable results, irrespective of the intent of treatment (curative or palliative).

PDT is an FDA-approved treatment and has a long history of clinical success in treating lung cancer. It involves systemic administration of the light-sensitive drug (i.e., photosensitizer), Photofrin^®^, which then preferentially accumulates in the target tissue and is cleared from surrounding normal tissues. After 48 h, target tissue is illuminated with laser light at a red wavelength of 630 nm, which activates Photofrin to generate toxic reactive oxygen species, notably singlet oxygen [6], and thus leading to tumor destruction. The process via which tumor is destructed is a combination of direct cellular and secondary vascular effects [7]. In addition to local tumor ablation, PDT can improve antitumor immunity as shown in pre-clinical and clinical settings. For NSCLC, PDT has been used historically as monotherapy or as combination therapy along with surgery, chemotherapy, or standard radiation therapy [5,12,13,14,15,16,17].

For early and advanced stage NSCLC, PDT has been approved for symptom management, curative and palliative intent, especially in cases with tumors that are ineligible for standard surgery and radiation therapy. Overall, severe toxicities are uncommon in PDT and it is well tolerated [5,12,16,17]. At the same time, in spite of its potential to be a promising treatment option, PDT remains underutilized mostly due to the lack of randomized trials comparing PDT and conventional therapies. 

Systemic therapy is viewed as the cornerstone of disease treatment in NSCLC patients with stage III or IV disease [4,18]. A randomized, prospective study among patients with oligometastatic NSCLC observed improved progression-free survival with local therapy (surgery or radiation), compared to systemic therapy alone [19]. However, PDT can be an option both for palliation and survival enhancement for many of these patients with symptomatic progression to airway compromise. Additionally, several of these patients may either not be candidates for or consent for chemotherapy. Radiation therapy alone may be employed in these cases, although it may not offer substantial rates or prolonged ability to generate disease control. This may be due to widespread disease or lack of ability to achieve ablative dose of radiation due to bulk of disease. In situations such as these, PDT may also offer enhanced and prolonged palliation to patients with airway obstruction. However, we know very little about how PDT can be incorporated with chemotherapy and radiation therapy. Therefore, in this study, we assess the association between PDT along with radiation and chemotherapy with radiation alone as reference group, among NSCLC patients with stage III or stage IV disease. Our hypothesis is that after adjusting for covariates, PDT demonstrates a survival benefit among patients with stage III or stage IV of NSCLC.

## 2. Results

### 2.1. Sample Characteristics

Between 2004 and 2016, 1,048,113 patients with NSCLC were identified, of which 667,527 patients had stage III or stage IV disease. Of these, 147 patients received PDT with radiation and chemotherapy, 227,629 patients received radiation with chemotherapy, 106,667 patients received radiation therapy alone and 122,193 patients received chemotherapy alone.

As shown in Table 1, radiation with chemotherapy group was younger (mean 63.6 years) compared to PDT group, radiation alone group and chemotherapy alone group (65.4, 68.3 and 66.3 years, respectively, *p* value < 0.0001). PDT group had lower proportion of whites, compared to radiation with chemotherapy, radiation alone and chemotherapy alone groups (79.6% vs. 83.2%, 82.5%, and 81.9%, respectively, *p* value < 0.0001). Almost half of the patients from the PDT group had at least one comorbidity (49.7%), compared to 33.5% of radiation with chemotherapy patients, 40.4% of radiation alone patients and 35.5% of chemotherapy alone patients (*p* value < 0.0001). Facility type differed across the four groups. For example, 41.5% of PDT patients were treated at academic programs, compared to 31.3%, 32.8%, and 33.9% from the radiation with chemotherapy group, radiation alone group, and chemotherapy group, respectively (*p* value < 0.0001). A higher proportion of PDT group patients had stage III disease (60.5%), compared to radiation with chemotherapy group, radiation alone group, and chemotherapy alone group (44.2%, 21.7%, and 20.7%, respectively; *p* value < 0.0001).

### 2.2. Overall Mortality

As reported in Table 1, overall mortality for PDT groups was 90.8%. This number was 87.9% for radiation with chemotherapy group, 95.6% for radiation alone group, and 91.3% for chemotherapy alone group (*p* value < 0.0001). The mean number of survival months was 18.6 for PDT group, 18.0 for radiation with chemotherapy group, 7.3 for radiation alone group, and 16.9 for chemotherapy alone group (*p* value < 0.0001). Multiple comparison using Tukey statistics showed that PDT group mean was different than that of the radiation alone group, and chemotherapy alone group. The mean for the radiation with chemotherapy group was also different from that of the radiation alone group, and chemotherapy alone group (all *p* values < 0.05).

### 2.3. Survival Analysis

**Figure 1:**
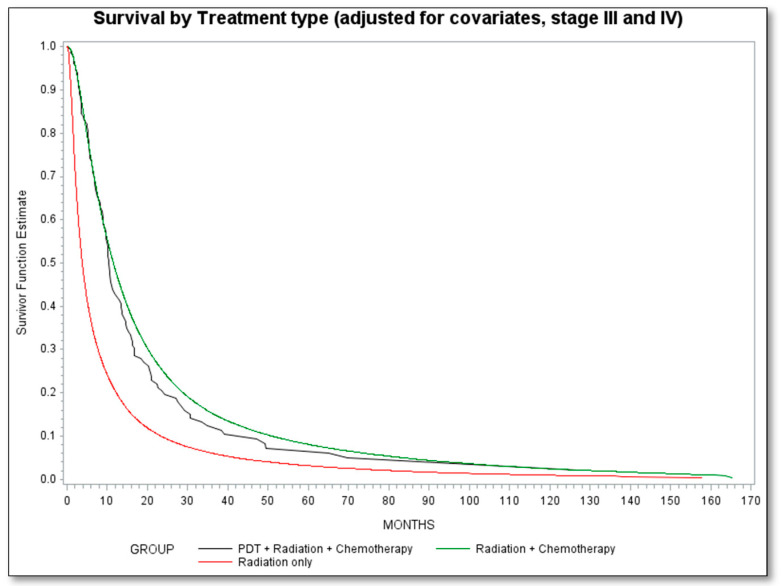
Survival by treatment type adjusted for covariates, stage III and IV NSCLC. Radiation is reference category.

Survival analyses evaluated the hazard of overall mortality and PDT exposure (Table 2) for NSCLC patients with stage III or stage IV disease. Compared to the radiation alone group, the PDT (with radiation and chemo) group and the radiation with chemotherapy group had lower hazard of overall mortality (50% lower and 53% lower, respectively; HR = 0.50, 95% CI = 0.42, 0.60; HR = 0.47, 95% CI = 0.46, 0.50, respectively). The adjusted survival curve is presented in Figure 1.

**Figure 2:**
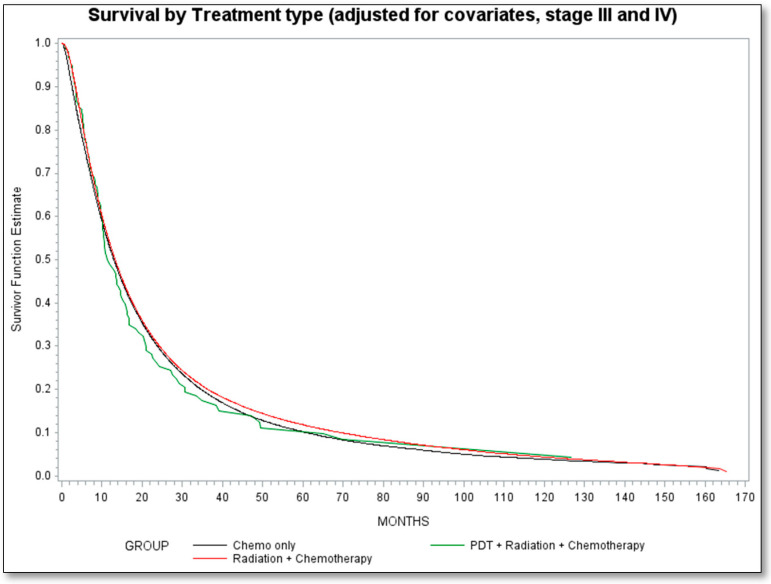
Survival by treatment type-adjusted for covariates, stage III and IV NSCLC. Chemotherapy is reference category.

We also observed that the hazard of mortality associated with PDT exposure was comparable with chemotherapy alone as the reference group as shown in Table 2 (HR = 1.03, 95% CI = 0.86, 1.23). A similar observation was made for the hazard associated with the radiation with chemotherapy group, compared to the chemotherapy alone group (HR = 0.98, 95% CI = 0.98, 1.02). The adjusted survival curve is presented in Figure 2.

## 3. Discussion

For advanced NSCLC, PDT is currently being used in combination with other treatment options such as chemotherapy, radiotherapy, and surgery. We observed that PDT, along with radiation and chemotherapy, is associated with enhanced overall survival among NSCLC patients with stage III or stage IV disease, compared to treatment with radiation alone. To our knowledge, this is the largest study that examines the association between PDT and mortality in a US cohort of patients with stage III or stage IV NSCLC. At the same time, PDT is more appropriate for NSCLC patients with endoluminal tumor growth represented by tumor infiltration of the trachea or the bronchi. Studies are underway to assess if PDT can reach tumors that are located in the peripheral lung parenchyma, and thus be a treatment for peripheral NSCLC.

For patients with advanced disease, PDT in part can offer prolonged maintenance of a patent airway, which in turn can enhance the quality of life. A large series recently showed that PDT, as a means to maintain airway patency, offers better outcomes compared with other interventional modalities such as YAG Laser, Argon Plasma Coagulation, and Cryotherapy [20]. Additionally, an up-regulation of the immune response that occurs with PDT helps in improving durability of tumor response and survival [21]. Especially advanced disease patients treated with PDT, chemotherapy and radiation therapy report most favorable outcomes in terms of disease control and survival advantage, thus emphasizing the need for multi-modal intervention in these patients. 

In general, the better outcomes associated with PDT may be due to local control that is more sustained. Alternately, similar to radiation therapy, this may be due to abscopal effect as a result of the immunogenic properties induced by PDT [22]. Studies have shown that local PDT treatment can result in systemic neutrophilia, induction of acute phase reactants, as well as systemic release of inflammatory cytokines [23]. Research has demonstrated that, in addition to the direct effects on tumor cell, PDT can enhance the antitumor immunity and thereby improve the tumor cell immunogenicity [22,23,24,25]. 

Certain disadvantages of PDT remain, such as complex scheduling, necessity of multiple procedures, higher initial cost due to Photofrin infusion, repeat bronchoscopies and inpatient observation following the procedure, and finally potential negative effects on patient’s quality of life due to photosensitivity issues during the weeks post-procedure [22,23,24,25].

Prior studies have reported beneficial outcomes of multimodal therapy (combination of local and systemic therapy) [26]. In a study of NCDB data of patients with stage IV NSCLC, surgical resection plus systemic therapy or EBRT/thermal ablation plus systemic therapy was shown to be beneficial compared to systemic therapy alone [27]. Among the selected patients with limited metastases, benefits of local therapy in conjunction with systemic therapy have been observed [28]. Studies in patients with oligometastatic NSCLC have shown the benefit of local consolidative therapy (surgical resection or chemo/radiotherapy) [19,29]. 

Results of a SEER–Medicare analysis showed that for stage III NSCLC, intensity modulated radiation therapy was associated with similar overall and cancer-specific survival and maintained similar toxicity risks compared with 3D-CRT [30]. Elderly patients with advanced NSCLC showed improved survival outcomes with use of chemotherapy [31]. In addition, for advanced NSCLC, adjuvant chemotherapy was associated with improved survival [32]. Multimodal therapy of systemic and local therapy (radiation or surgery) showed improved survival [3,33,34,35]. However, many patients diagnosed with NSCLC are elderly have medical comorbidities, poor functional status or notable preexisting weight loss and may not be able to tolerate chemotherapy with radiation. Patients who refuse or are not candidates for chemotherapy or surgery often receive radiation therapy alone. A recent NCDB study showed that hypofractionated radiation therapy is a viable option for locally advanced NSCLC [36]. Given the heterogeneity of the NSCLC, many systemic and local therapy options are available. However, optimal management for patients with T3 and T4 NSCLC remains unclear.

An earlier randomized controlled study compared the safety and efficacy of PDT plus external beam radiation vs. external beam radiation alone among 41 patients with inoperable non-small cell bronchogenic carcinoma obstructing a central airway. It was noted that, compared to radiation alone, the addition of PDT prior to radiation therapy resulted in significantly favorable and longer lasting local control of the disease [11]. In another study, the safety and effectiveness of combined brachytherapy and PDT were evaluated in patients with bulky endobronchial lung cancer. Of the 32 patients in the study, 15 were technically inoperable and 17 had recurrent bronchogenic carcinomas. The complete histological response rate of PDT with brachytherapy was 97%, leading to the conclusion that the combination of PDT with brachytherapy was safe with strong therapeutic efficacy in this group of patients with lung cancer [10]. Additional clinical studies have also demonstrated the effectiveness of PDT as a palliative therapy to relieve airway obstruction resulting from NSCLC.

Our study has several limitations. First, our study uses secondary data and consists of persons who were at least 18 years of age. We did not have data related to smoking status, pulmonary function tests, tumor location and type of chemotherapy. 

Performance status is an important measure of the effects of tumor symptoms, with other comorbidities, on a patient’s day-to-day function, such as ability to self-care. However, in the NCDB database, this information is not available. At the same time the Charlson–Deyo comorbidity index score, which is a good predictor of mortality, is available and we have adjusted our analysis using this score. Thus, our comparison between treatments can be considered as exploratory, and future prospective studies using performance status data can validate our findings.

While usefulness of NCDB in examining cancer diagnosis and primary cancer treatment is well documented, it must be noted that NCDB is not a population-based database, as NCDB cases are identified from the hospitals where they present for cancer diagnosis and/or its treatment. At the same time, NCDB captures about 75% of all incident cancers in the United States, and thus has an advantage over other cancer registry data. Additionally, NCDB includes more comprehensive data on certain treatments such as chemotherapy [37]. Despite these limitations, PDT appears to offer improved disease control and survival benefit for advanced NSCLC patients. It should be considered more often as part of multidisciplinary thoracic oncology care.

## 4. Materials and Methods

### 4.1. Data Sources and Study Cohort

We used retrospective data from the National Cancer Database (NCDB). The NCDB is a national hospital-based cancer registry that is co-sponsored by the American College of Surgeons (ACoS) and the American Cancer Society. Established in 1989, NCDB houses data from more than 1500 hospitals with ACoS-accredited cancer treatment programs, accounting for almost 70% of all newly diagnosed cancer cases in the United States with accumulated data on 29 million cancer cases. Records of patients with biopsy-proven non-small cell lung cancer diagnosed between January 2004 and December 2016, and with stage III or stage IV disease were obtained from the NCDB. The analyses were conducted between 1 January 2019 and 31 December 2019, and all data were de-identified. This study was approved by the University of Pennsylvania institutional review board.

### 4.2. Measurements

#### 4.2.1. Overall Mortality

Key dependent variable for the study was overall mortality and was obtained from NCDB’s vital status variable, which is status of the patient at the last contact. Time to death was defined as number of days between date of diagnosis and date of death. Patients who were alive at last contact were censored. 

#### 4.2.2. Treatment Type

From the NCDB database, we first created a cohort of patients whose treatment was coded as “Laser ablation or cryosurgery” or as “Local tumor destruction or excision, NOS” (which includes PDT). From Medicare and Medicaid claims between 2000 and 2013, we identified those NSCLC patients who received PDT with Photofrin (Jcode: J9600) and also those who received bronchoscopy (CPT code 31641); we then used these to confirm PDT treatment. Next, from the NCDB database, we extracted groups of NSCLC patients who received radiation with chemotherapy, radiation therapy alone or chemotherapy alone. 

#### 4.2.3. Covariates

We used sociodemographic, disease severity, and medical comorbidity data from NCDB to adjust our measures of association between PDT treatment and overall mortality. Sociodemographic variables were age, race, gender, insurance status, income, and education. Disease severity was measured using cancer grade and stage, and the T and N categories were based on clinical assessment. Medical comorbidity was measured using Charlson–Deyo comorbidity score. Charlson–Deyo score is an age-independent, comorbidity score index used to predict long-term survival. It is mapped from as many as ten reported ICD-9-CM or ICD-10 diagnosis codes (comorbidities) typically from discharge abstracts or billing face sheets [38,39,40,41].

It must be recognized that treatment for NSCLC is not randomly assigned, and socio-demographic characteristics can affect the type of treatment that a patient receives. Therefore, we determined the propensity (i.e., probability) of receiving PDT treatment as a function of socioeconomic characteristics. We then adjusted our analysis for propensity score, along with other covariates. 

### 4.3. Statistical Analysis

To begin with, we used appropriate tests (χ^2^ and *t*-test) to compare the sociodemographic characteristics, disease severity and comorbidity. In all analyses, a two-sided *p* < 0.05 was considered statistically significant. Survival analysis was used to study the association between treatment groups and overall mortality. The key independent variable was exposure to PDT, with radiation therapy alone as reference category. We also carried out similar analysis with chemotherapy alone as the reference category. The models were adjusted for sociodemographic characteristics (age, gender, race and ethnicity, education, income, geographic area, insurance status), disease severity (cancer stage), medical comorbidity, and propensity score. We used Statistical Analysis System (SAS), Version 9.2 (SAS Institute, Cary, NC, USA), for analysis.

## 5. Conclusions

Future research in NSCLC patients with stage III or stage IV disease can identify the pathways via which PDT helps in improving patient survival. In addition, the impact of this association on the health-related quality of life among NSCLC patients needs to be addressed. In summary, our hospital registry-based study spanning up to 12 years following the diagnosis of stage III or stage IV NSCLC shows that PDT, in combination with other systemic therapy and local therapy, was associated with significantly improved overall survival. Thus, providers need to assess the incremental benefits of PDT in NSCLC patients with stage III or stage IV disease. 

## Figures and Tables

**Table 1:** Socio-demographic and clinical characteristics comparison (stage III/IV).

Covariates	PDT (+Radiation + Chemo) *n* = 147, *n* (%)	Radiation + Chemo *n* = 227,629, *n* (%)	Radiation *n* = 106,667, *n* (%)	Chemo *n* = 122,193*n* (%)	*p* Value
Mean age (std)	65.4 (8.9)	63.6 (10.3)	68.3 (11.1)	66.3 (10.9)	<0.0001
Male gender	92 (62.6)	128,376 (56.4)	60,373 (56.6)	66,123 (54.1)	<0.0001
Race					<0.0001
White	117 (79.6)	189,487 (83.2)	87,967 (82.5)	100,119 (81.9)	
African American and other	28 (20.4)	381,42 (16.8)	187,00 (17.5)	22,074 (18.1)	
Insurance status					
Not Insured/Other	11 (6.4)	15,261 (6.7)	8082 (7.6)	6523 (5.3)	<0.0001
Private/MCO	44 (30.3)	84,061 (36.9)	24,489 (22.9)	39,924 (32.7)	
Medicaid/Medicare	92 (63.5)	128,307 (5642)	74,096 (69.5)	75,746 (61.9)	
Education *					<0.0001
≥29%	31 (21.1)	43,236 (19.1)	22,303 (21.0)	23,007 (18.9)	
20% to 28.9%	35 (23.8)	59,710 (26.3)	28,134 (26.5)	29,909 (24.6)	
14% to 19.9%	38 (25.9)	56,024 (24.7)	25,507 (24.0)	29,535 (24.3)	
<14%	43 (29.3)	67,774 (29.9)	30,284 (28.5)	39,223 (232.2)	
Income					<0.0001
Less than USD 38,000	19 (12.9)	36,425 (16.1)	19,201 (18.1)	18,607 (15.3)	
USD 38,000–USD 47,999	27 (18.4)	45,079 (19.9)	21,963 (20.7)	22,900 (18.8)	
USD 48,000–USD 62,999	45 (30.6)	65,545 (28.9)	30,556 (28.8)	34,021 (27.9)	
USD 63,000+	56 (38.1)	79,632 (35.1)	34,432 (32.4)	46,126 (37.9)	
Geographic area					0.3093
Metro	115 (78.2)	179,826 (79.0)	84,350 (79.1)	99,092 (81.1)	
Non metro	32 (21.8)	53,803 (21.0)	22,317(20.9)	23,101 (18.9)	
Cancer program					<0.0001
Community /Comprehensive	59 (40.1)	124,324 (55.2)	56,788 (53.5)	65,351 (54.1)	
Academic/Research	61 (41.5)	70,605 (31.3)	34,823 (32.8)	40,960 (33.9)	
Integrated Network	27 (18.4)	30,438 (13.5)	14,627 (13.8)	14,555 (12.0)	
Charlson/Deyo score					<0.0001
0	74 (50.3)	151,490 (66.6)	63,469 (59.9)	78,834 (64.5)	
1	46 (31.3)	53,722 (23.6)	27,624 (25.9)	29,722 (24.3)	
≥2	27 (18.4)	22,417 (9.9)	15,574 (14.5)	13,637 (11.1)	
Stage					<0.0001
III	89 (60.5)	100,507 (44.2)	23,185 (21.7)	24,503 (20.1)	
IV	58 (39.5)	127,122 (55.9)	83,482 (78.3)	97,690 (79.9)	
Histology	40 (27.2)	95,874 (42.1)	43,011 (40.3)	63,637 (52.1)	<0.0001
Adeno Carcinoma	19 (12.9)	42,921 (18.9)	22,503 (21.1)	19.905 (16.3)	
Non-Small Cell Squamous Cell	74 (50.3)	61,996 (27.2)	26,066 (24.4)	20,936 (17.1)	
Other	14 (9.6)	26,838 (11.8)	15,087 (14.2)	17,715 (14.5)	
Mortality	*n* = 130	*n* = 209,390	*n* = 98,534		
Overall	118 (90.8)	183,956 (87.9)	94,239 (95.6)	103,265 (91.3)	<0.0001
Mean time to death in months (std)	18.6 (23.9)	18.0 (21.1)	7.3 (12.6)	14.7 (16.9)	<0.0001

* Proportion of adults in the patient’s zip code who did not graduate from high school.

**Table 2:** Survival analysis stage III and IV (overall survival), propensity score adjusted.

Covariates	Hazard Ratio (HR), 95% Confidence Interval (95% CI)	Hazard Ratio (HR), 95% Confidence Interval (95% CI)
Treatment		-
PDT (+radiation + chemo)	0.50 (0.42, 0.60)	
Radiation + chemo	0.47 (0.46, 0.50)	
Radiation only (Ref)	-	
Treatment	-	
PDT (+radiation + chemo)		1.03 (0.86, 1.23)
Radiation + chemo		0.98 (0.98, 1.02)
Chemo therapy only (Ref)		-
Age at diagnosis	1.02 (1.01, 1.04)	1.03 (1.01, 1.06)
Gender		
Male	1.19 (1.17, 1.21)	1.24 (1.21, 1.26)
Female (Ref)	-	-
Race		-
White	1.32 (1.29, 1.35)	1.37 (1.34, 1.40)
African American	1.22 (1.19, 1.25)	1.28 (1.24, 1.31)
Other (Ref)	-	
Insurance status		
Not Insured/Other Government	1.01 (0.99, 1.05)	1.04 (1.01, 1.08)
Private/Managed Care	0.92 (0.90, 0.96)	0.92 (0.89, 0.97)
Medicaid/Medicare (Ref)	-	-
Income		
Less than USD 38,000	1.02 (1.01, 1.07)	1.06 (1.04, 1.09)
USD 38,000–USD 47,999	1.04 (1.02, 1.09)	1.06 (1.01, 1.08)
USD 48,000–USD 62,999	1.03 (1.01, 1.10)	1.04 (1.02, 1.10)
USD 63,000 + (Ref)	-	-
Urban		
Metro	0.99 (0.97, 1.04)	0.99 (0.96, 1.05)
Urban	1.02 (0.99, 1.08)	1.01 (0.97, 1.07)
Rural (Ref)	-	-
Charlson–Deyo score	1.15 (1.12, 1.18)	1.17 (1.15, 1.21)
Stage		
III	0.55 (0.52, 0.59)	0.62 (0.58, 0.68)
IV (Ref)	-	-

## Data Availability

Publicly available datasets were analyzed in this study. These data can be found here: https://www.facs.org/Quality-Programs/Cancer/NCDB (accessed on 1 January 2019), link/accession number.
